# The effect of Dr Google on doctor–patient encounters in primary care: a quantitative, observational, cross-sectional study

**DOI:** 10.3399/bjgpopen17X100833

**Published:** 2017-05-17

**Authors:** Noor Van Riel, Koen Auwerx, Pieterjan Debbaut, Sanne Van Hees, Birgitte Schoenmakers

**Affiliations:** 1 GP and Researcher, Public Health and Primary Care, University of Leuven, Leuven, Belgium; 2 GP and Researcher, Public Health and Primary Care, University of Leuven, Leuven, Belgium; 3 GP and Researcher, Public Health and Primary Care, University of Leuven, Leuven, Belgium; 4 GP and Researcher, Public Health and Primary Care, University of Leuven, Leuven, Belgium; 5 GP and Professor of General Practice, Public Health and Primary Care, University of Leuven, Leuven, Belgium

**Keywords:** primary care, patient empowerment, patient education, health care

## Abstract

**Background:**

Two-thirds of all patients search the internet prior to a health consultation.

**Aim:**

To explore how searching for online health information before visiting a doctor influences patients’ behaviour during the consultation.

**Design & setting:**

A quantitative, observational, and cross-sectional study of 18–75-year-old patients who used the internet.

**Method:**

Patients were recruited by social media for the quantitative study. This was followed by a qualitiative study of GPs who were questioned in focus groups. Two questions were addressed: What is the effect of searching online health information on the behaviour of the patients? How does the GP handle this information?

**Results:**

Almost half of all responders (total *n* = 963) usually went to the doctor after the online information search but two-thirds were not reassured by the internet search. More than half of responders had more confidence in their GP after searching online. The older the responders, the more they went to the doctor after their internet search and the younger the responders, the more they were worried. The more frequently people consulted the internet for specific complaints, the more likely they reported reassurance.

**Discussion:**

Patients usually made an appointment with their GP after the internet search. New symptoms are rarely noticed and the search usually did not lead patients to distrust their GP. The majority of GPs described positive effects of the online search behaviour on the consultation.

**Conclusion:**

The emerging use of the internet for searching health information, commonly referred to as 'Dr Google', is not seen as a threat by GPs and leads to a better mutual understanding of symptoms and diagnosis.

## How this fits in

More patients are searching the internet before consulting their GP and they not only search for an online answer to symptoms but also to prepare for a consultation. GPs believe that this does not undermine the doctor–patient encounter and well-informed patients contribute in a positive way to the consultation.

## Introduction

Health information is an increasingly accessible topic to the more than 3.2 billion people who have access to the internet worldwide.^1^ More than half of Belgian people (56%) use the internet, anecdotally known as 'Dr Google', to gather health-related information.^2^ Those who search the internet for information about illness and health are mainly young, female, and highly educated.^3–5^ However, two-thirds of people find this information unreliable and one-third report confusion after the search^6–7^ People search for information about diseases, symptoms, and treatments and also general health information.^8–9^ One-fifth look up information to facilitate communication with their doctor^3,10^ and also seek information for their relatives.^11–12^ The sources that patients use are diverse: websites of support groups, patient blogs, websites created by editors of popular media, and websites of (para)-medical organisations and health professionals.^13–14^ The effect of this online information search on the behaviour and feelings of patients has been poorly investigated.^9,11^

Medical doctors, including GPs are not very familiar and confident with patients who 'Google'.^5,15^ When questioned about how they deal with this phenomenon, doctors indicated that they feel threatened and that their professional expertise had been disregarded. Therefore, doctors tend to ignore or contradict the e-information patients bring to the consultation. Moreover, in academic and professional networks, there is a tendency to discourage patients to search for online health-related information.^16–17^

This study primarily explored how searching for online health information before attending a general practice consultation affects people’s actions, behaviour, and feelings. The study also examined if the effects of this online search behaviour on GPs and how this influences the consultation and doctor–patient relationship.

## Method

### Study design and population

First, a descriptive and exploratory study was conducted to answer the following research question: What is the effect of searching online health information prior to a general practice consultation on the actions, behaviour, and feelings of the population?

The study population was recruited in public and non-public places (private GP practices) in Flanders and on social media. Only Flemish citizens aged 18–75 years old were enrolled and those who had never used the internet or did not use the internet to look up health-related information were excluded. To obtain reliable results, at least 385 included responders were required. This sample size was calculated based on a margin of error of 5%, a confidence interval (CI) of 95%, a population in Flanders of 6 million people and a standard deviation of 50%.

The study design was quantitative, observational, and cross-sectional. A survey was drawn up to determine personal characteristics, the frequency of internet use, the frequency of searching online health information, and frequency of doctor visits, followed by 12 statements which asked about the impact of online search behaviour on the actions, behaviour, and feelings of the patients. The contents of the questionnaire and of the statements were determined by the results of a preceding literature review and refined through discussion in the research group in attendance of practicing GPs. The survey was distributed online through social media (Facebook^®^) and email during the period of March and April 2016. In addition, on 4 and 8 April 2016, in street surveys were conducted.

A Likert scale was used to measure the answers to the 12 statements: never, not usually, usually, always, no opinion/I don’t know. The last option was added to avoid random responses. The outcome variables were the tendency to go to the doctor, the tendency for self-medication, concerns about the complaints, the perception of the pre-existing problems, the awareness of new complaints, and confidence in the GP.

The second part was a descriptive, exploratory study in a qualitative design to answer the secondary research question: whether and to what extent the effects of searching online health information prior to a general practice consultation are noticed by the GP and if they have any influence on the consultation.

For this purpose, seven statements were discussed with GPs who worked in Flanders and had a least 2 years of experience. The GPs were contacted by phone and email and asked to participate in the study. Five statements were developed based on the results of the survey from the first part of the study. Two statements measured the effect of the online search behaviour on the GP consultation and how online search behaviour influenced the consultation. The seven statements were adjusted in successive phases and resubmitted in accordance to the Delphi method.^15^

These seven statements were discussed individually with four GPs in May 2016. The responses from these first discussions were analysed according to the common ground technique and adjusted where required. These modified statements were then discussed with five other GPs, also in May 2016. For both rounds the answers to the statements were compiled to one result per statement. The researches chose this unconventional approach of the Delphi interview technique to enlarge the input of the experts (in this case the GPs).^18^ These experts (*n *= 9) all belonged to the same professional network and were chosen carefully to compose a representative sample (by age, sex, practice type, geographical features). 

The final statements served as a guide for discussion on the subject.

### Data analysis

A descriptive analysis was run on the set of demographics and responses to the survey questions. Next, a multiple regression analysis was performed. The independent ordinal variables were age, education level, the frequency of internet usage, the frequency of searching online health information, and frequency of GP consultations. The 12 statements of the population were the dependent ordinal variables. The analysis was executed with the statistical computer program SAS (version 9.4) in which logistic regression was used. The coefficient of determination *R²* was calculated to determine to what extent the variability in responses could be explained by the independent variables. Non-standardised regression coefficients were considered statistically significant if the corresponding *P*-value was <0.05.

The discussions with the GPs were led by the researchers, recorded verbatim, and analysed by the Delphi method.

## Results

### Patient survey

#### Description of population

Nine hundred and sixty-three people participated in the survey. Ninety-five surveys were incomplete and 15 responders were excluded since they did not use the internet. A further 135 responders were excluded as they never used the internet to look up health information and, in total, 718 responders met the inclusion criteria (Table 1).

**Table 1.** Population characteristics

Variable (*n *= 718)	*n*			%
Sex Male Female	238 480			33.15 66.85
Age Mean age, years (SD) 18–25 26–35 36–45 46–60 61–75	40	201 123 122 182 90	(15)	27.99 17.13 16.99 25.35 12.53
Education level Primary school Middle school High school College Professional	7 59 203 241 208			0.97 8.22 28.27 33.57 28.97
Frequency of internet use Monthly Weekly Daily More than once a day	1 18 237 462			0.14 2.51 33.01 64.35
Frequency of internet use to gather health information Usually not Usually Always	308 323 87			42.90 44.99 12.12
Frequency of GP consultations Never Less than once a year One to several times a year Monthly More than once a month	15 152 512 36 3			2.09 21.17 71.31 5.01 0.42

Note: Some variables may not add to 100% due to of rounding.

The survey was mainly completed by female patients (66.85%, 480/718). The average age of responders was 40 years (SD = 15). One-third of responders graduated high school (28.27%, 203/718), more than one-third obtained a college (undergraduate) degree (33.57%, 241/718), and nearly one-third a university (postgraduate) degree (28.97%; 208/718). The majority of responders (97.36%, 699/718) used the internet at least once a day. Three-quarters of responders (76.74%, 551/718) visited a GP at least once a year.

### Effects of searching online health information on actions, behaviour, and feelings

Almost half of responders (45.71%, 288/630) usually went to the doctor after the online information search (Table 2). More than one-tenth (13.11%, 78/595) usually did not go to the doctor although they had planned to before the search. More than one-quarter (26.37%, 159/603) consulted the GP sooner than they had intended to do before the search. The majority of responders (70.65%, 467/661) never self-medicated irrespective of the search and after the search more than one-tenth (13.37%, 73/546) thought there was no need for medication. Two-thirds (59.56%, 358/601) were not reassured after the search and one-third (29.81%, 189/634) were worried after the search. After the search more than one-tenth of responders (12.38%, 78/630) felt that their symptoms were worse but more than half of responders (56.84%, 295/519) had more confidence in their GP after searching online.

**Table 2.** Responders’ answers to the statements: 'After my online search to gather health information …'

Statements	Never	Usually not	Usually	Always
	*n* (%)	*n* (%)	*n* (%)	*n* (%)
I go to the GP (*n = *630)	61 (9.68)	211 (33.49)	288 (45.71)	70 (11.11)
I do not go to the GP although this was the original plan before the search (*n *= 595)	248 (41.68)	265 (44.54)	78 (13.11)	4 (0.67)
I am more likely to visit the GP than I intended before the search (*n = *603)	132 (21.89)	312 (51.74)	144 (23.88)	15 (2.49)
I start medication without consulting a doctor (*n = *661)	467 (70.65)	151 (22.84)	40 (6.05)	3 (0.45)
I do not think I need medication although I did think this before the search (*n = *546)	204 (37.36)	269 (49.27)	70 (12.82)	3 (0.55)
I am reassured (*n = *601)	74 (12.31)	284 (47.25)	226 (37.60)	17 (2.83)
My worries about my symptoms increase (*n = *634)	116 (18.30)	329 (51.89)	174 (27.44)	15 (2.37)
I have the feeling my symptoms got worse (*n = *630)	254 (40.32)	298 (47.30)	69 (10.95)	9 (1.43)
I have the feeling my symptoms got better (*n = *595)	206 (34.62)	314 (52.77)	69 (11.60)	6 (1.01)
I notice additional complaints I did not notice before the search (*n = *630)	226 (35.87)	299 (47.46)	98 (15.56)	7 (1.11)
I have more confidence in my GP than before the search (*n = *519)	93 (17.92)	131 (25.24)	201 (38.73)	94 (18.11)
I have less confidence in my GP than before the search (*n = *596)	329 (55.20)	239 (40.10)	23 (3.86)	5 (0.84)

Note: Some variables may not add to 100% due to rounding.

### Link between population characteristics and the effects of online searching behaviour

There was no statistically significant influence of the independent variables (age, education level, the frequency of internet usage, the frequency of searching online health information and frequency of GP consultations) on reporting increased severity of symptoms *(F* = 1.60, *P* = 0.1572) (Table 3). There was also no association between these population characteristics and reporting increased confidence in the GP (*F* = 1.61, *P* = 0.1543).

**Table 3.** The correlation between the independent variables and the effects of online searching: multiple regression analysis.

Variable	F	R^2^	Age category, *b*	Degree, *b*	Frequency of internet use, *b*	Frequency of internet use for health information, *b*	Frequency of GP visits, *b*
GP^1^	11.42^a^	0.08	0.07^b^	0.02	0.09	0.01	0.39^a^
Not going to the GP anymore^2^	4.88^b^	0.04	0.03	0.01	−0.01	0.18^a^	−0.12^c^
More likely to go to the GP^3^	3.42^b^	0.03	0.03	−0.05	−0.03	0.10^c^	0.11
Self-medication without advice^4^	5.54^a^	0.04	−0.01	0.03	0.09	0.12^b^	−0.08
No medication anymore^5^	4.72^b^	0.04	−0.02	−0.01	0.05	0.19^a^	−0.06
Reassurance^6^	4.67^b^	0.04	0.03	−0.01	−0.04	0.18^a^	−0.09
Increased anxiety^7^	9.50^a^	0.07	−0.06^b^	0.002	0.04	0.19^a^	0.22^a^
Increased severity of symptoms	1.60	0.01	−0.02	−0.01	0.09	0.05	0.06
Decreased severity of symptoms^9^	2.76^c^	0.02	−0.03	−0.002	−0.03	0.15^b^	−0.01
Additional symptoms^10^	4.48^b^	0.03	−0.04	0.03	0.05	0.12^b^	0.15^b^
More confidence in the GP^11^	1.61	0.02	0.04	−0.03	−0.08	−0.05	0.10
Less confidence in the GP^12^	5.31^a^	0.04	0.01	−0.03	0.03	0.18^a^	−0.02

*b* = regression coefficient. ^a^ *P*<0.0001. ^b^ *P*<0.01. ^a^ *P*<0.05.

Statements 'After my online search for health information …':

*^1^ I go to the GP*

*^2^ I do not go to the GP although this was the original plan before the search.*

*^3^ I am more likely to visit the GP than I intended before the search.*

*^4^ I start medication without consulting a doctor.*

*^5^ I do not think I need medication although I did think this before the search.*

*^6^ I am reassured.*

*^7^ My worries about my symptoms increase.*

*^8^ I have the feeling my symptoms got better.*

*^9^ I have the feeling my symptoms got worse.*

*^10^ I notice additional complaints I did not notice before the search.*

*^11^ I have more confidence in my GP than before the search.*

*^12^ I have less confidence in my GP than before the search.*

There was no statistically significant association between the level of education and the effects of online search behaviour (*P>*0.05), nor was there a statistically significant association between the frequency of internet use and the effects of online search behaviour (*P>*0.05).

The older the responders, the more they went to the doctor after their internet search (*b *= 0.07, *P *= 0.0070). The younger the responders, the more they were worried after a search on the internet (*b *= −0.06; *P *= 0.0061).

Responders who frequently searched online health information often decided not to go to the doctor (*b *= 0.18; *P*<0.0001). They also took medication more often without the advice of the GP (*b *= 0.12, *P *= 0.0011). The more frequently people consulted the internet for specific complaints, the more likely they reported reassurance (*b *= 0.18; *P*<0.0001). At the same time there was also an increase in anxiety (*b *= 0.19; *P*<0.0001). The number of responders who developed additional complaints increased (*b *= 0.12, *P *= 0.0045). At the same time, there was an increase in the number of responders who reported a decrease in severity of symptoms (*b *= 0.15; *P *= 0.0004). The more frequently responders used the internet concerning health issues, the less confidence they had in their GP after the online information search (*b *= 0.18; *P*<0.0001).

Responders who frequently visited the GP more often went to a GP consultation after their search on the internet (*b *= 0.39; *P*<0.0001). Patients who frequently consult their doctor more often presented with an increase in anxiety (*b *= 0.22; *P*<0.0001) and noticed additional complaints after the online search (*b *= 0.15, *P* = 0.0065).

#### GP Survey

Based on the results of the population survey, seven statements were generated (Table 4). In a first round these statements were discussed with four GPs. After this discussion an analysis of the interviews was made and the statements were adapted.

**Table 4.** Statements discussed with the GPs. A: statement based on the descriptive analysis of the survey’s results, B: adjusted statement based on the first four conversations with GPs

**Statement 1** A. Most of my patients do not start self-medicating after their online search to gather health information. B. Most of my patients do not start self-medicating after their online search to gather health information. If they do so, they do it mainly when they have benign symptoms or on the advice of significant others. **Statement 2** A. The online search for health information does not influence the confidence of the patient in their GP. B. The online search for health information does not influence the confidence of the patient in their GP. **Statement 3** A. The online search for health information does not make my patients experience additional symptoms. B. The online search for health information does not make my patients experience additional symptoms. Some people will be more likely to do so, such as patients in their 20s and 30s and patients with tendencies toward hypochondria. **Statement 4** A. The online search for health information does not influence the severity of my patient’s symptoms. B. The online search for health information does not influence the severity of my patient’s symptoms. **Statement 5** A. I do not have the feeling that the online search for health information makes my patients more anxious. B. I do not have the feeling that the online search for health information makes my patients more anxious, although naturally anxious people often become more anxious after the search. Moreover, this is probably the group of patients that searches the most on the internet for health information. **Statement 6** A. If I know that my patient searched online to gather health information, this will complicate the consultation. B. If I know that my patient searched online to gather health information, this will not influence the consultation in a negative way. First of all, there is the possibility that we will come together to a broader differential diagnosis. Secondly, I can easily respond to the patient’s ideas, concerns, and expectations and patients will hesitate less to ask for specific diagnostic tests. This can lead to an interesting dialogue. **Statement 7** A. If patients tell me about the information they found on the internet, I can discuss this during the assessment and plan. For example, I can give them websites where they can find health information in the future. B. If patients tell me about the information they found on the internet, I will put that information in perspective. For example, I can give them websites where they can find health information in the future.

On statement 1, the GPs unanimously agreed that the majority of patients did not start self-medicating after an online search. According to three GPs, opinions of relatives added more weight to the decision of self-medication than the online search. Additionally, the GPs supposed that when there were serious symptoms, patients rarely self-medicated.

On statement 2, seven out of nine GPs did not have the feeling that the internet search affected confidence in the GP. They assumed that if patients continued visiting the practice, there were no confidence issues. As one said: ‘a doctor merits his patients’. On the other hand, two GPs argued that is was very difficult to estimate the level of confidence patients have in their doctor.

On statement 3, eight GPs, thought patients rarely experienced additional symptoms after a search on the internet. Seven GPs expected that patients with a more hypochondriac profile experienced more symptoms after the search. Two GPs noticed that it was very intrusive and indiscrete to ask patients if symptoms worsened after the search. Two GPs reported that the information seekers with worsened symptoms were in particular those patients aged 20–30 years old.

On statement 4, the GPs unanimously agreed to the argument that the internet search does not affect the severity of symptoms.

On statement 5, a small majority of GPs (five out of nine) believed that the internet search made their patients more anxious. The other four GPs stated that anxiety was rather inherent to the patient and the context: ‘At this time we see many more worried pregnant women than in the recent past’. All GPs agreed that concerns increased more in naturally anxious patients: 'The anxious patient only reads what could happen.'

On statement 6, the majority of physicians (eight out of nine) described positive effects of the online search behaviour on the consultation. The GPs could easily capitalise on the ideas, concerns, and expectations of patients. They indicated that the research work of the patient sometimes made a contribution to the diagnosis, and that sometimes they even learned something new. One older GP, who stilled operated without computer, argued that using this patient health information complicated the consultation. He planned a new consultation after he verified the information the patient presented.

On statement 7, most GPs (eight out of nine) indicated that explaining the obtained information and putting it into perspective was very important. One of the doctors insisted: 'You may never ridicule the information the patient found online.' The GPs also mentioned the importance of providing reliable website references to their patients. The older GP still worked with paper leaflets to inform patients on health-related topics.

## Discussion

### Summary

This study explored the effects of searching online health information before attending a general practice consultation on the actions, behaviour, and feelings of the Flemish population. The study showed that people usually make an appointment with their GP after the internet search. Most people said the search did not make them more worried. More than 80% of responders experienced no difference in severity of symptoms after the online information search. New symptoms are rarely noticed and the search usually does not lead patients to distrust their GP.

### Strengths and limitations

One of the strengths of this study is a large number of enrolled responders. The trends in the results of the screening and the GP survey are also very similar.

One of the weaknesses of the study is the low significance of the results. The variability in the effects of online searching can only be explained partially by the independent variables. Possible bias in this study is not excluded: a selection bias which is reflected in the asymmetric distribution of sex and education and a possible information bias since responders remarked during survey administration that some statements were rather difficult to interpret. As this study may not give causal links, a controlled experiment could be set up in the future.

A further weakness is the low number of participating GPs and the unusual approach of the Delphi Interview technique. Indeed, two groups of GPs were interviewed in two consequent rounds. The first group was asked to discuss and reflect on the statements, the second group was presented the adapted statements. A plea in favour of this strategy is the careful selection of GPs: the sample of GPs was composed based sociodemographic and practice-related features (even a GP without computer participated as well as a fully digitally-equipped group practice). Both groups were matched based on these features. The decision to work with different groups for both interview rounds added expertise and insight. The statements were not structurally adapted after round one but rather refined and more nuanced for a better understanding.

### Comparison with existing literature

More than half of Flemish internet users in this study who search online health information consulted their doctor, with a tendency toward the older generation and those who frequently visit the doctor. More than 30% usually did not visit the doctor after the internet search. These numbers are confirmed by previous research.^4,10,12^ The fact that more than 40% of people rarely or never visit a doctor after the online search could be explained by the low threshold to consult the internet for health problems. The internet can provide the patient with a better idea of the need for a doctor visit.^9–10,19^ Individuals who frequently use the internet to search health information changed their initial plan most often. This may be explained by the presumption that they are more exposed to unreliable information that alters their understanding of symptoms and increases the need for reassurance. The GPs therefore stress the importance of a better visibility of reliable health information websites. This way patients will better be able to frame their complaints and to determine the urge of seeking medical help.

This research showed that after the online search for health information people rarely started medication without consulting a doctor. This trend has been extrapolated by Santana *et al*.^9^ The Flemish GPs indicated that they noticed most patients do not start self-medicating. If this is done anyway, it is usually well-known or over-the-counter medication. Many patients, however, do not realise that this is also medication and hence did not report its use which may partly cause the low numbers. The GPs also stated that an online search usually did not alter the severity of symptoms or the confidence patients have in their doctor. The ﻿doctor–patient relationship appears to be strong enough to resist the impact of questionable health information.

This study suggested that the online search usually did not make people more worried about symptoms. The Flemish doctors concurred that it is particularly the anxious patient who seeks online information and consequently remains even or more anxious afterward. This is consistent with the finding that those who frequently use the internet for health information are worried after the search. In particular young people and those who frequently go to the doctor were anxious after searching online. The latter seems logical, because anxious patients probably tend to go to the GP more often. GPs also mentioned that information seeking behaviour depended on the context. Healthcare issues such as pregnancy, obesity, heart diseases, diabetes, and cancer affect many people and are therefore popular subjects of searches and of concerns.^3,6,8,12^ The GPs suggested that patients with tendencies toward hypochondria experience additional symptoms.

Somewhat unexpectedly, this study did not show a significant association between educational attainment and concerns about symptoms after searching online. This could be explained by the fact that those with fewer years of education have less access to the internet and therefore were under-represented in the study population.

The severity of symptoms appeared to be slightly influenced by the online search for health information. The interviewed GPs confirmed this observation. Furthermore, only 17% of responders noticed additional symptoms after the online search. This result is rather surprising since it is assumed that an online search leads to additional complaints. GPs believed that in particular the 20–30 years old were prone to an increase of symptoms. This can be explained by their internet use: they are not yet the digital natives but neither digital illiterate which means that their strategies might be inadequate.^13,20^

However, the research showed that those individuals who frequently use the internet for health problems more often noticed additional symptoms.

Only 5% of the population had less confidence in the GP after the online search for health information. The research showed that the more a patient uses the internet to look up health information, the less they trust the GP. This apparent contradiction can be explained by bias or an inversed causal relationship: patients who intensively consult internet might not have a trusted GP (yet).

Hu *et al* and Iverson *et al *noted earlier that confidence in the physician was not affected by the online search behaviour of the patient.^5,10^ The interviewed GPs perceived this similarly. It was previously shown that patients using the internet as a health information source still state that the doctor is the best source of information.^6,15^ The GPs added that 'they get the patients they deserve': allowing the patient to actively participate in the consultation using health information, ensures the mutual confidence.^20^

Finally, the majority of surveyed GPs were very positive towards the online search behaviour of their patients. It facilitates the dialogue with patients. Obviously, GPs have to pay attention to the source of information and the type of patient doing the research. Doctors could verify the information together with the patient and refer them to trustworthy websites.^8,17,21^ The one GP without internet access during consultation even planned a new consultation after he verified the patient information. In the internet era, it is a responsibility and a task of the medical schools to teach students to deal with health-seeking patients.^22–23^

### Implications for research and practice

The emerging use of the internet for searching health information, commonly referred to as Dr Google, is not a threat for the Flemish GP. The large majority of the Flemish population would still visit the doctor after online information retrieval and the patient’s confidence in their GP is rarely affected. On the contrary, it may lead to a better mutual understanding of symptoms and diagnosis. Therefore, an open attitude toward the patient and their online search behaviour is recommended. GPs often overestimate the possible anxiety caused by an online information search.

In the future, further research could examine how vulnerable groups deal with online health information and in which way they are at risk. Second, medical schools need to teach students how to deal with information-seeking patients and how to benefit from this trend as a practising doctor. Third, medical doctors and authorities have a major responsibility in guiding patients to reliable websites.

In conclusion, at a time in which shared decision making and patient empowerment is the norm for conducting good medical practice, Dr Google has a generally positive contribution to the GP consultation and the doctor–patient relationship in Flanders.
